# The effects of transformational leadership dimensions on employee performance in the hospitality industry in Malaysia

**DOI:** 10.3389/fpsyg.2022.913773

**Published:** 2022-09-16

**Authors:** Brenda Ern Wei Teoh, Walton Wider, Abidah Saad, Toong Hai Sam, Asokan Vasudevan, Surianti Lajuma

**Affiliations:** ^1^Faculty of Business and Communications, INTI International University, Nilai, Negeri Sembilan, Malaysia; ^2^Faculty of Business Management, Universiti Teknologi MARA (UiTM), Merbok, Kedah, Malaysia

**Keywords:** employee performance, transformational leadership, idealized influence, individualized consideration, inspirational motivation, intellectual stimulation, hospitality industry

## Abstract

Employee performance plays a crucial role in the productivity of organizations, especially in the hospitality industry in Malaysia. This work performance is influenced by leadership style, and finding the type of leadership style that is suitable to apply to employees is crucial, especially in the midst of the COVID-19 pandemic. Transformational leadership theory is selected for this study in determining leadership styles. There are four dimensions under transformational leadership theory, namely idealized influence, individualized consideration, inspirational motivation and intellectual stimulation. Data were collected online from 400 employees working in the hospitality industry in Malaysia and analyzed using partial least square structure equation modeling (PLS-SEM). The findings show that only two dimensions of transformational leadership, namely idealized influence and inspirational motivation, have a significant positive influence on employee performance. Implications in the context of human resource management and recommendations to increase employee performance are also discussed.

## Introduction

The COVID-19 pandemic had impacted employee performance, and organizations must take proactive action to improve work performance (Wiradendi Wolor et al., 2020). In 2020, the COVID-19 pandemic and the Movement Control Order (MCO) had a detrimental impact on employee performance and mental health, resulting in substantial employee turnover (Shanmugam et al., 2020). According to Qualtrics’ newest Asia Pacific Employee Pulse Study, Malaysia has the greatest job satisfaction in the region, with 67% job satisfaction, despite high levels of stress and lengthy working hours (Business, 2017b). However, according to a Gallup poll, only 15% of the world’s workers are engaged in their jobs, with 87% of Malaysian workers disengaged (Wiradendi Wolor et al., 2020). According to the Covid-19 Epidemic Employee Pulse, 74% of Malaysian workers worked from home as a result of MCO, which proved ineffective as 77% of workers lost productivity, and just 23% maintained performance levels (Haroon, 2020). When working from home in Malaysia, poor employee performance was caused by slow internet speeds, difficulty accessing company systems, and a lack of company resources (Shanmugam et al., 2020).

The hospitality industry in Malaysia, in particular tourism, can be said to be a thriving business, and quality employee performance is crucial to sustain it. According to Zubair and Shamsudin (2021), tourism can generate revenue from tourists very quickly, as it involves travel expenses, lodging services expenses, hotel accommodation expenses, cost of food, and tourists’ destination costs. As tourism is such a massive business, it creates opportunities for professional growth, and Malaysia has seen incredible growth in the tourism sector. It also opens up the possibility of earning foreign currency (Karim et al., 2020). Furthermore, Malaysia (2021) reported that the gross output value of lodging services was RM15.8 billion in 2017, up from RM13.9 billion in 2015, representing a 6.7% annual growth rate. In keeping with the rise in the value of gross output, the value of intermediate input grew by RM0.8 billion to RM6.8 billion, representing a 6.2 percent annual growth rate, resulting in a value added of RM9.0 billion in 2017. The number of people employed in this industry increased by 3.3 percent to 139,410 in 2016, up from 130,675 in 2015. Meanwhile, wages and salaries paid in 2017 totaled RM3.5 billion, up from RM3.0 billion in 2015. It is obvious that hospitality workers help contribute enormously to this sector, and quality work performance is essential to continuously prop it up.

Despite the fact that the COVID-19 pandemic is likely to have an impact on employee performance (Wiradendi Wolor et al., 2020), little attention has been paid to whether the types of leadership behavior or style influence employee performance in the hospitality industry in Malaysia during the pandemic. Shapoval et al. (2021) asserted that the study of hospitality crisis management is essential for two reasons. The first is that the global economy is shifting from a manufacturing economy toward a service industry. To begin, an excessive dependence on the profits generated by the hospitality business may be disastrous since a decline in those profits adds to a general negative trend in the economy as well as an increase in the number of people looking for work. The second reason is that the way an industry conducts its recovery has a huge effect in how well that industry recovers from a crisis. The crisis management skills of an organization should be of such a high level that a developing crisis may be addressed swiftly and prevented from spreading to the greatest degree feasible. Because the COVID-19 pandemic still on-going, its entire influence on the worldwide hospitality sector from the standpoint of leadership and its possible implications need to be recognized. While earlier research on the impact of leadership style on employee performance (Kahya, 2018; Nagarathinam, 2020) has been substantial and found to have a significant impact, the studies had been conducted under normal conditions. Focusing on leadership during this crisis is important not only because it is relatively under-explored, but also because it carries important social implications and organizational costs (Lombardi et al., 2021). Especially considering that the hotel industry is one of the sectors that has been impacted the most severely by the COVID-19 crisis all over the world, the dynamics and leadership requirements of the industry deserve further attention (Hahang et al., 2022). Since the detrimental impact on overall employee performance is obvious, it is necessary to determine whether leadership style will still have a substantial impact on employee performance under stressful COVID-19 conditions (Shanmugam et al., 2020).

It can be said that leadership style has a positive and substantial effect on employee performance (Pawirosumarto et al., 2017). This is because it plans, directs and develops a positive working environment for employees (Alwedyan et al., 2017). Employee performance is positively influenced by effective leadership style because individuals reach their full potential in a company where leaders create a favorable environment by coaching, monitoring, offering guidance, supporting, and rewarding appropriately (Nagarathinam, 2020). The function of leadership is critical in leading to an increased performance from employees to achieve the organization’s objectives (Ibrahim and Daniel, 2019). The effectiveness of employee performance suffers when firms do not pay attention to the value of leadership (Helmold, 2020). Leaders are role models who emphasize high morals and integrity, lead by example, and are compassionate and altruistic, hence inspiring colleagues to perform (Bai et al., 2019). According to the transformational leadership theory by Burns (1978) and Bass (1985), transformational leaders stimulate their subordinates to form new perceptions of leadership as a result of intellectual stimulation, and leaders are able to form perceptions as individuals capable of supporting and caring for their subordinates with individualized consideration, thanks to inspirational motivation and charisma. Employees are content to work and perform under transformational leadership styles, because they are coached, monitored, encouraged and rewarded based on their performance level (Nagarathinam, 2020).

Leadership that is solely concerned with the company’s profits has a poor relationship with employees, which leads to poor employee performance (Quade et al., 2020). Leaders must provide a conducive working environment, as well as motivate and inspire employees to voluntarily achieve job objectives set by the organization, and leaders must provide adequate technological facilities and support, as well as ample training to increase employees’ knowledge and skills in order to improve performance (Nagarathinam, 2020). Studies on the role of leaders in connecting with employees in the workplace are often disregarded (Busse and Regenberg, 2019). Therefore, the purpose of this research is to determine the elements of transformational leadership that impact employee performance in the hospitality industry, replicating previous studies (Buil et al., 2019; Şeşen et al., 2019). With better understanding, employers can apply transformational leadership theory to improve employee performance (Krisnanda and Surya, 2019). While there has been previous research (Kalsoom et al., 2018; Priarso et al., 2018; Şeşen et al., 2019; Top et al., 2020), more study is needed on the particular processes *via* which transformational leadership positively impacts employee performance, as well as the boundaries within which transformational leadership increases employee performance (Buil et al., 2019). Therefore, this research topic is ideal in order to provide valuable insights into the relationship between transformational leadership and employee performance in the hospitality industry in Malaysia.

It is anticipated that the findings in this study will pave the way for the management team of accommodation service providers under study to have a clear picture on how transformational leadership affects employee performance within the hospitality industry of Malaysia. This investigation will help to provide a deeper understanding on employee performance and create awareness of the challenges that employee performance presents to organizations in the Malaysian hospitality industry. Subsequently, it will effectively help organizations and managers to tackle employee performance. Many academics have looked into the topic of leadership styles, but the material available is still limited as the many distinct leadership styles have varying effects on employees or job satisfaction (Ibrahim and Daniel, 2019). According to Ahmad et al. (2020), 60% of studies on Transformational Leadership discuss employee-related outcomes, with only 10% of the outcomes being related to employee performance. Factors such as management support, training culture, organizational climate, job environment, job communication, job autonomy, proactivity, adaptability, and intrinsic motivation are widely discussed as predictors of employee performance (Diamantidis and Chatzoglou, 2019; Rozi and Sunarsi, 2020). Despite the fact that numerous research has discovered that transformative leadership has a direct or indirect impact on employee performance, it is difficult to locate a study that explores how different dimensions of transformational leadership are related to employee performance (Ferozi and Chang, 2021). Additionally, employee performance is a subject that is widely studied around the world, and is also popular among Malaysian scholars (Basri et al., 2017; Lor and Hassan, 2017). However, there have been few studies with regard to leadership styles practiced under pandemic circumstances (Kuofie and Muhammad, 2021). As a result, there are gaps in research that justify conducting research on such a topic.

## Literature review

### Theoretical underpinnings

The foundation for the study is the transformational leadership theory proposed by Burns (1978) and further developed by Avolio and Bass (1995). Transformational leadership can foster positive employee behavior as it is seen to play an essential role in shaping effective management (Buil et al., 2019). As it can change the organization’s strategy, mission, structure and culture to drive product and work innovation, followers and organizations may benefit greatly from transformational leadership (Mustika et al., 2020). The way a company or organization operates reflects the extent to which the leader advances the company’s development and recognizes the role of employees or subordinates (Hasib et al., 2020). Work performance gives organizational benefits that arise from transformational leadership (Buil et al., 2019). Transformational leaders will inspire followers to pursue their own goals, establish performance standards, and provide constructive criticism, as well as assist followers in becoming more creative and imaginative, and pay attention to their requirements. Leadership is an aspect of an organization’s management that has a substantial influence on its performance and success (Kalsoom et al., 2018). Transformational leadership is more than just directive; it is also concerned with the followers’ performance and growth. These leaders will strengthen the bond between their followers and themselves by raising their morale in order to better manage the organization by way of encouraging, inspiring and enabling their employees to achieve organizational success through their work. It can also persuade followers to generate high-quality work, which can lead to economic achievements (Hasib et al., 2020). Furthermore, transformational leaders will pay attention to each employee’s unique demands and professional backgrounds, as well as provide opportunities for people to grow in a sustainable manner (Jiang et al., 2017). All of these behaviors will enhance relationships and benefit the work performance of employees.

Transformational leaders are able to inspire their followers because of four unique but interrelated behavioral components namely (i) idealized influence, which focuses on ethics, morals and trust; (ii) inspirational motivation, in which leaders provide significance and challenge to their followers’ work, employing inspirational messages to elicit emotions; (iii) intellectual stimulation, whereby leaders support new ways of thinking by questioning existing assumptions, ideas and traditions, as well as emphasizing the significance of problem-solving skills and the use of logic; and (iv) individualized consideration, which refers to leaders who evaluate the needs, abilities, and aspirations of their followers and give the required coaching and mentoring.

### Employee performance

Employee performance suffered during the COVID-19 pandemic in 2020, as businesses struggled to keep track of and balance the workloads of employees who worked from home and in the workplace (Wiradendi Wolor et al., 2020). During the shutdown in Malaysia, employees suffered mental health difficulties, which resulted in a drop in employee performance (Shanmugam et al., 2020). It is critical at this time to understand each employee because organizational decisions are made based on the job performance of each individual, which leads to organizational success (Alromaihi et al., 2017). Employee performance has been shown to have a favorable impact on organizational productivity when employees are well-led, innovative, open to new ideas and resource-efficient (Mohamed et al., 2018).

Employee performance is vital in the manufacturing business, which contributed 22.8 percent to Malaysia’s GDP and is the second greatest contributor among other industries (Business, 2017a). Job satisfaction and employee performance will suffer from workplace pressures, a lack of motivation, low compensation and bad leadership, resulting in low productivity in Malaysian businesses (Shanmugam et al., 2020). According to a poll performed by Regus, a worldwide workspace supplier, 53 percent of Malaysians work from home, which is higher than the global average of 48 percent. However, this arrangement has resulted in a progressive reduction in performance (Haroon, 2020). In order to perform and contribute high productivity to a firm, the type of job must match the employee’s personality, skill, experience, knowledge and wage expectations, and recruiting policy is critical in matching job with employee qualities (Shanmugam et al., 2020). According to a Jobstreet (2017)'s poll, Malaysia ranks fourth among seven ASEAN countries, in terms of employee satisfaction, with leadership being the key determinant in preventing happiness at work. According to a poll conducted by the Malaysian Employers Federation, small and medium-sized companies (SMEs) in Malaysia have a 22 percent yearly employee turnover rate, owing to inadequate recruitment policies that result in low job satisfaction (Business, 2017a). It is vital for employers in Malaysia to provide better resources to their employees in order for them to perform to their full capacity, such as fast Internet, pleasant office space, training and superior computers, all of which improve job satisfaction and productivity (Shanmugam et al., 2020). The study of employee performance, therefore, is critical as it determines organizational productivity, which could have an impact on Malaysia’s economy. Though there have been academic studies earlier on employee performance, this study is a descriptive attempt to learn more about employee performance, specifically in the hospitality industry of Malaysia during the COVID-19 pandemic.

### Transformational leadership style and employee performance

The term “transformational leadership” was coined by (Burns, 1978) and (Bass, 1985) to describe the impact of exceptional leaders on subordinates’ responses, as well as the method by which leaders build rapport with followers, attend to specific needs, and help followers grow and thrive. Idealized influence, inspirational motivation, intellectual stimulation and individualized consideration are four characteristics of transformational leadership that can change individual interests into common interests in order to achieve more than what is intended (Alrowwad et al., 2017). Acts of individualized consideration, according to Lai et al. (2020), can provide support to members who are afraid of a negative outcome if they present their true selves at work. According to these early definitions, transformational leadership is a leadership style in which “leaders and followers push one other to a greater degree of moral and motivation” (Burns, 1978). Transformational leaders, according to Lai et al. (2020), establish holistic and communal goals for their followers and persuade them that these goals are worthwhile. The term “transformational” comes from the word “transform,” which means “to convert from one state to another” (Hijazi et al., 2017). Transformational leadership is a leadership style in which the leader promotes and motivates subordinates to innovate and create change in order to grow and impact the future success of the company, hence exceeding original performance goals (Choi et al., 2017). Yukl (1989) reaffirms this by stating that transformational leadership is defined as conduct that transforms individuals and organizations toward achieving a long-term objective by adjusting to changes in social, physical, environmental or psychological best practices. Conversely, transformational leadership is utilized by a leader who wants a team to gain more power and work outside of the current status quo in order to achieve a whole new set of business objectives (Bernarto et al., 2020).

Yukl (1989) emphasized the importance of persuading others to carry out a request, support a proposal, and make a choice when followers are driven to follow the leader’s instructions. This is also reinforced by Bourne and Smith-Sherwood (2018), who claim that an excellent leader may stimulate and massage his or her followers’ cognition into action by harmonizing people’s perceptions. The next attribute is power, which underlies a leader’s understanding of how to influence the company’s followers (Hassan et al., 2019). Transformational leadership is a critical component impacting employee performance and affective commitment (Astuty and Udin, 2020). This remark is further supported theoretically and empirically by Li et al. (2019), who found that transformational leadership is linked to improved employee performance.

### Idealized influence

One of the dimensions of transformational leadership is idealized influence (Nidadhavolu, 2018). Influencing is the process of attempting to transform people’s attitudes, behaviors, thought patterns, beliefs, goals, needs, values, abilities, and actions in order to achieve specific objectives (Wirawan, 2017). A transformational leader motivates, stimulates and inspires his followers to accomplish extraordinary outcomes (Lindawati and Parwoto, 2021). Because transformational leaders display determination and a sense of authority while providing a clear vision and sense of mission to their followers, they are able to acquire the trust and respect of their subordinates or followers (Widodo et al., 2020). Idealized influence, or “charisma,” according to Buil et al. (2019), denotes leaders that uphold high moral and ethical standards, are self-assured, have high personal esteem and act as excellent role models for their followers. Leaders impact members’ conduct in the workplace because they are viewed as a typical example of the organization and have the authority to assess members’ performance or make choices concerning their advancement, which may influence members’ behavior (Lai et al., 2020).

According to Langat et al. (2019), employee job performance among lower level insurance company managers in Kenya was positively affected by idealized influence. Transformational leaders serve as mentors in their teams or departments, focusing on the followers’ personal development, learning and accomplishments (Nidadhavolu, 2018). As influence is actively used by someone against others to shape activities and relationships inside an organization, transformational leadership has a major positive impact on employee performance (Nugroho et al., 2020). It has a strong beneficial effect on job performance, indicating that the leader’s huge support will be able to provide great motivation to employees to work better (Bastari et al., 2020). Besides that, under varied degrees of epidemic crisis perception, moral modeling and charisma have a substantial positive correlation with crisis management performance (Ma and Yang, 2020). Thus, we hypothesized that:

*Hypothesis 1*: Idealized influence positively affects employee performance in the hospitality industry in Malaysia.

### Individualized consideration

Individualized consideration is another characteristic of transformational leadership in which the leader pays special attention to and treats each employee individually (Widodo et al., 2020). As a result, transformational leaders can improve employee development since they will coach and advise their subordinates in different ways depending on their needs (Nidadhavolu, 2018). Transformational leaders may successfully change the status quo in their firm by practicing proper behavior at each level of the transformation process because it goes beyond solely trade or admiration for the performance displayed by followers. It is built on trust and dedication rather than on only trade or gratitude for followers’ performance (Delegach et al., 2017). Transformational leadership is a process that forecasts the value of employees by developing individuals to achieve set goals (Alrowwad et al., 2017). Transformational leaders can approach their followers as individuals, taking into account their individual needs, desires and skills, as well as displaying concern for each individual follower, thereby assisting them in developing their own abilities, and spending time leading and educating them individually (Busari et al., 2020). Wilson (2020) backs this up, stating that transformational leadership relies on charisma, intellectual stimulation and individualized evaluation of the requirements of followers to help people reach their full potential. Leaders engage with followers to build a bond of relationship through individualized consideration (Martin, 2017). Individual goals and needs are communicated to subordinates, and they work together to attain their professional goals (Fauzi et al., 2021).

Individualized leadership consideration encourages subordinates to exhibit positive beliefs in upholding their strengths, proactive support and the development of constructive behaviors (Chen et al., 2018). According to Bastari et al. (2020), the more creative the leader guides the employee, the better the employee’s job performance. It fully mediates between interactional justice and affecting employee performance if each employee receives a sense of pleasure from the personalized consideration received from leadership and management (Khan et al., 2020). Besides that, employee satisfaction rises dramatically when leaders devote special attention to each employee’s development needs, thus forming a personal bond with them. These leaders serve as counselors or trainers, catering to followers’ distresses and needs on an individual basis (Khalil and Sahibzadah, 2017). According to Okafor and Egboka (2021), employers should monitor and engage with subordinates in order to determine their distinct personalities, which will aid in the use of tailored consideration methods when assigning duties and responsibilities to them. Therefore, we hypothesized that:

*Hypothesis 2*: Individualized consideration positively influences employee performance in the hospitality industry in Malaysia.

### Inspirational motivation

The third characteristic of transformative leadership is inspirational motivation (Alrowwad et al., 2017). Employees communicate with transformational leadership by setting high expectations for how they perceive the ideal future to be (Nugroho et al., 2020). Inspirational motivation expresses the aim in simple terms and expands on it based on the workforce’s demands (Hashim, 2019). This trait of transformational leadership typically leverages emotional qualities and pushes subordinates to motivate them to work hard to meet the organization’s objectives (Alrowwad et al., 2017). Inspirational motivation energizes individuals by expressing a motivating and exciting vision, and transformational leaders motivate followers to share a goal and empower them to achieve it (Buil et al., 2019). Transformational leadership is defined as a motivator who connects with his or her employees by understanding their needs and encouraging them to work for the company’s long-term goals through the presentation of a clear vision (Berraies and Bchini, 2019). According to Lai et al. (2020), transformational leaders provide meaningful goals, move members’ concerns from self-interests to communal aims, and create a safe and supportive environment that encourages followers to focus on current responsibilities. The company gains from transformational leadership since it is able to understand its employees’ requirements in order to retain them by managing workload in a friendlier manner, resulting in subordinates feeling satisfied with their work (Al-Khaled and Fenn, 2018).

According to numerous literatures, transformational leadership is the most successful leadership style in the firm (Nidadhavolu, 2018; Berraies and Bchini, 2019; Nugroho et al., 2020) and transformational leaders focus on maximizing human capabilities and improving trust relationships by influencing their behavior. Transformational leadership instills inspiration, motivation and a collective sense of mission in employees, making them feel motivated and aspirational with a heightened awareness of goal (Hansen and Pihl-Thingvad, 2019). In the Iraqi Kurdistan region, transformational leaders’ inspirational motivation had a favorable effect on employee performance (Top et al., 2020). Kinya and Eliud (2021) found that top managers pushed subordinate staff to achieve more by articulating high expectations that subordinate staff aspired to fulfil and encouraging optimism. To increase employee performance in regulatory state firms, leaders should be committed to the organization’s mission by speaking optimistically about future goals and collaborating with employees to move people forward with inspiring words and actions (Anyiko-Awori et al., 2018). Besides that, according to Hasija et al. (2019), a leader who emanates inspirational motivation can have a big impact on how their employees participate in institute-related activities and contribute to the institution’s growth and survival. Hence, we hypothesized that:

*Hypothesis 3*: Inspirational motivation positively influences employee performance in the hospitality industry in Malaysia.

### Intellectual stimulation

Intellectual stimulation is a term that has been defined as the pursuit of intellectual, reasoned and careful problem-solution action based on the capacities of the followers (Hashim, 2019). A transformational leader is more likely to serve as a role model for their subordinates, showing them how to approach an issue from a new or different viewpoint or come up with more innovative solutions (Samson and Ilesanmi, 2019). Nugroho et al. (2020) reported that transformational leaders enable their subordinates to build a sense of participation and self-determination by bringing creative chaos or thinking outside the box. While inspirational motivation energizes followers by articulating a motivating and exciting vision, and transformational leaders inspire followers to share a vision and empower them to achieve it, intellectual stimulation refers to leaders who encourage followers’ creativity by presenting challenging new ideas and different ways to solve problems (Buil et al., 2019). The transformational leader typically presents a future vision to drive staff to build new problem-solving strategies, and is open to listening to employee complaints and demands (Hoxha, 2019). As employees are not restricted or compelled to follow any rules or guidelines when executing the assignment, a leader with these characteristics will surely operate as a trainer, advisor or consultant who creates a positive working environment for them (Herminingsih and Supardi, 2017). According to Tawas (2019), transformational leadership aims to transfer followers to a new level of demand and aspiration by transforming their values and self-concepts. The transformational leader always encourages employees to be aware of the output, to prioritize the needs of the group, and to uphold the greater demands of employees in order to push them to accomplish work performance that exceeds the company’s expectations (Nugroho et al., 2020).

**Figure 1:**
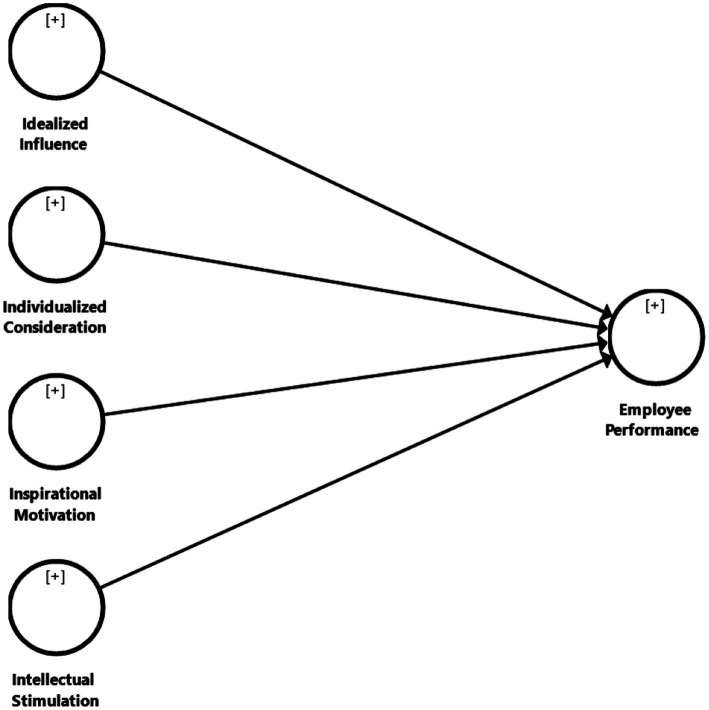
Research Framework.

Leaders who practice intellectual stimulation can motivate their followers to be imaginative and innovative in dealing with old difficulties in new ways (Busari et al., 2020). Subordinates’ interest in problem-solving, as well as their capacity and competency to think in new and creative ways, can be increased by leaders who can stimulate their intelligence (Carreiro and Oliveira, 2019). These leaders may empower their followers by boosting their willpower and confidence, which will boost their cognitive abilities to solve issues in novel ways (Al-Husseini and Elbeltagi, 2018). Employee performance is positively associated to intellectual stimulation, which suggests that when leaders inspire their followers, the followers will improve their performance (Dialoke and Ogbu, 2018). Ayacko et al. (2017) reported that employees’ performance was greatly influenced by intellectual stimulation, thus supervisors should be creative in performing job obligations, encourage creativity in solving work-related difficulties, and value employees who are curious and want to learn more. There is also a link between employee performance and creativity culture, and researchers recommend that managers provide employees the freedom to be creative in terms of time and space, as well as being in a supportive atmosphere that recognizes their needs (Hankir et al., 2020) (refer to Figure 1). Hence, we hypothesized that:

*Hypothesis 4*: Intellectual stimulation positively influences employee performance in the hospitality industry in Malaysia

## Method

### Population and sample

The study population is identified as employees working in 4 and 5 star hotels located in Malaysia. Only those four and five-star hotels which are registered under Malaysian Association of Hotels are considered. The 4- and 5-star hotels were selected because of their strong international visitor occupancy rate and willingness to share data. The total estimated number of employees in the accommodation service industry of Malaysia is 130,397 (Malaysia, 2019). We conducted an online survey and recruited 400 participants using a convenience sampling method. To assess the minimum required sample size in terms of statistical power, we used G*Power software (Faul et al., 2009). The model of this study had four predictors. By using G*Power with an effect size of 0.15, alpha of 0.05, and a power of 0.95, the minimum sample size needed was only 129. Thus, we can safely say that our study with a sample size of 400 has a power of more than 0.95 and is large enough, and the findings can be used with confidence.

### Data collection procedure

Data collection was held over a two-week period in December 2021. This study on human participants did not require ethical approval because it was carried out in accordance with the Declaration of Helsinki and academic ethics. To participate in this study, participants provided written informed consent. To ensure compliance with safety requirements, switching to online data collection was deemed necessary. Using a convenience sampling technique, the participant fulfilling the inclusion criterion was selected to complete the self-report survey administered *via* the Internet, through e-mail and WhatsApp. A total of 400 responses were received from respondents, of which all responses were usable. The response rate for the study was 100%. Table 1 demonstrates the demographic information of the respondents of whom 50.7% were male and 49.3% were female. Majority of the respondents came from the 21–30 age group at 47.3%, followed by 31–40 at 41.0%, 41–50 at 9.4%, and 51 and above at 2.3%. Regarding working experience, majority had been working in their respective companies for 1–5 years at 48.3%, followed by less than 1 year, at 29.0%, 11 years and above, at 12.7%, and 6–10 years, at 10.0%. In terms of job position, most of the participants were in rank and file, at 34.0%, followed by executive, at 27.0%, manager, at 24.8%, and head of department, at 14.2%.

**Table 1:** Demographic profile of respondents.

		Frequency	%
Gender	Male	203	50.7
	Female	197	49.3
Age	21–30 years old	189	47.3
	31–40 years old	164	41.0
	41–50 years old	38	9.4
	51 years old and above	9	2.3
Work experience	Less than 1 year	116	29.0
	1–5 years	193	48.3
	6–10 years	40	10.0
	11 years and above	51	12.7
Job position	Rank & File (e.g., F&B Attendant, Front Office Assistant)	136	34.0
	Executive	108	27.0
	Manager	99	24.8
	Head of Department	57	14.2

### Measures

#### Employee performance

This construct comprises 3 items that are adapted from Na-Nan et al. (2018) using the 5-Point Likert Scale, ranging from 1 (*strongly disagree*) to 5 (*strongly agree*). The following items were used: “I perform tasks attentively and correctly”; “I always complete my work assignments on schedule”; and “My work is up to the standard of my employer.” The Cronbach alpha coefficient was.85.

#### Transformational leadership

This construct comprises 16 items adapted from Avolio and Bass (1995) using the 5-Point Likert Scale, ranging from 1 (*strongly disagree*) to 5 (*strongly agree*). There are four dimensions in this scale, namely: idealized influence (e.g., “my superior talks about his/her most important values and beliefs”); inspirational motivation (e.g., “my superior talks optimistically about the future”); individualized consideration (e.g., “my superior spends time teaching and coaching me”); and intellectual stimulation (e,g., “my superior seeks differing perspectives when solving problems”). The Cronbach alpha coefficient ranged from.72 to.93.

### Data analysis

In this study, we used PLS-SEM as the statistical method to examine the research model, utilizing SmartPLS 3.3.3. PLS-SEM is fundamentally used to develop theories in exploratory research. Therefore, we employed PLS-SEM due to the inherent suitability of this approach for exploratory studies, which is the objective of this study (Ali et al., 2018). PLS-SEM incorporates the assessment of the measurement model (outer model) and structural model (inner model; Anderson and Gerbing, 1988). In order to assess the measurement model, the relationships between latent variables and their measurement items or indicators will be evaluated (Ali et al., 2018). In addition, the adequacy of the measures used in the current study will also be examined through indicator reliability and validity. The method includes checking the reliability of individual indicators, the internal consistency reliability of each construct, and convergent and discriminant validity (Hair et al., 2017). For the structural model assessment, path coefficient analysis is performed to explain the relationship between each latent variable. The model examines the endogenous variables (R^2^), the estimation of the path coefficient and a confidence interval of 95% (CI 0.95; Hair et al., 2017).

### Justification using SEM-PLS

Although PLS-SEM has been used for exploratory purposes since its outset, many researchers have discovered that it can also be used for confirmatory or exploratory purposes (Ringle et al., 2020). The PLS-SEM method appears to be accepted in many journals or publications for confirmatory purposes because it employs an established theory for testing (Afthanorhan et al., 2020).

## Results

### Measurement model assessment

This section demonstrates the criteria necessary to affirm convergent validity, discriminant validity, and construct reliability of the measurement model. This study comprised five reflective constructs, namely, employee performance, idealized influence, inspirational motivation, individualized consideration, and intellectual stimulation. In establishing construct reliability, the value of Composite Reliability (CR) and Cronbach’s alpha (CA) should be greater than 0.70. Two items (IS2 and IM4) were deleted due to multicollinearity issue. The values for CR and CA in this study ranged between 0.856 and 0.956, thus confirming construct reliability. Next, to assess convergent validity, the threshold value of average variance extracted (AVE) and outer loadings should be more than 0.5 (Ali et al., 2018). The results of the AVE in this study ranged between 0.666 and 0.879, thus meeting the required thresholds. Furthermore, the outer loadings values which ranged between 0.646 and 0.961, were considered acceptable as they were above 0.50. Thus, it can be concluded that all constructs indicated an acceptable degree of convergent validity therefore. The complete results are shown in Table 2.

**Table 2:** Results of measurement model assessment.

Latent variable	Items	Loading	AVE	CR	CA	Mean	*SD*
Employee performance	EP1	0.914	0.7543	0.900	0.856	4.56	0.58
	EP2	0.749					
	EP3	0.928					
Idealized influence	IF1	0.934	0.718	0.910	0.868	4.30	0.90
	IF2	0.743					
	IF3	0.758					
	IF4	0.934					
Inspirational motivation	IM1	0.945	0.819	0.931	0.891	4.33	0.66
	IM2	0.81					
	IM3	0.952					
Individualized consideration	IC1	0.809	0.666	0.887	0.888	4.35	0.68
	IC2	0.961					
	IC3	0.817					
	IC4	0.646					
Intellectual stimulation	IS1	0.951	0.879	0.956	0.936	4.38	0.73
	IS3	0.931					
	IS4	0.93					

Discriminant validity alludes to the actual degree of distinction between a certain construct and other constructs (Hair et al., 2017). It shows that one latent variable is novel in the model and can present phenomena that other constructs cannot. To ascertain discriminant validity, Heterotrait-Monotrait (HTMT) ratio was used in this study. When the correlation between each pair of latent exogenous constructs is less than 0.90, discriminant validity is achieved for HTMT (Henseler et al., 2015). Table 3 shows that the HTMT value for all constructs is less than 0.90, confirming discriminant validity.

**Table 3:** Discriminant validity using HTMT ratio.

No	Constructs	1	2	3	4	5
1	Employee performance					
2	Idealized influence	0.335				
3	Individualized consideration	0.243	0.848			
4	Inspirational motivation	0.303	0.763	0.742		
5	Intellectual stimulation	0.256	0.756	0.81	0.875	

### Structural model assessment

As seen in Table 2, the mean scores and standard deviations (*SD*) for our study variables were 4.56 for employee performance (*SD* = 0.58); 4.30 for idealized influence (*SD* = 0.90); 4.35 for inspirational motivation (*SD* = 0.74); 4.35 for individualized consideration (*SD* = 0.68); and 4.32 for intellectual stimulation (*SD* = 0.76). The collinearity between research variables was examined to ensure that the structural model did not include any lateral collinearity issues (Hair et al., 2017). Table 4 shows that all inner VIF values were below 5 (Hair et al., 2017), indicating that collinearity among the predictor constructs was not a serious issue in the structural model. The path coefficients of the structural model were examined using the bootstrap method with 5,000 resamples as suggested by Hair et al. (2017). The results show an *R*^2^ value of 0.154 for employee performance, which is considered substantial for behavioral science studies (Hair et al., 2019).

**Table 4:** Results of hypothesis testing.

Hypothesis	Relationship	Coefficient	*t*-value	95% CI	Supported	VIF
H1	IF → EP	0.239	2.386	[0.037, 0.408]	Yes	2.95
H2	IC → EP	0.103	0.624	[−0.097, 0.499]	No	3.13
H3	IM → EP	0.187	2.158	[0.056, 0.362]	Yes	2.89
H4	IS → EP	−0.097	0.728	[−0.421, 0.103]	No	3.91

EP = Employee Performance; IF=Idealized Influence, IC=Individualized Consideration; IM = Inspirational Motivation; and IS=Intellectual Stimulation.

**Figure 2:**
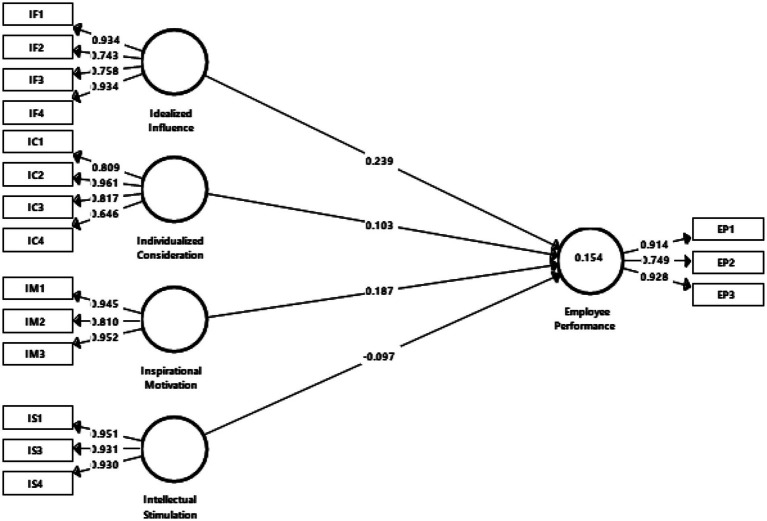
Results of assessment of structural model.

Next, the path coefficients in relation to the model’s latent variables were evaluated. Table 4 shows the results of the hypotheses testing, including the path coefficients for each path. The results show that idealized influence (*β* = 0.239, *t* = 2.386, *p* < 0.05), and inspirational motivation (*β* = 0.187, *t* = 2.158, *p* < 0.05) positively influenced employee performance, thus supporting hypotheses H1 and H3. Meanwhile, individualized consideration (*β* = 0.103, *t* = 0.624, *p* > 0.05) and intellectual simulation (*β* = −0.097, *t* = 0.708, *p* > 0.05) did not show a significant positive influence toward employee performance, thus hypotheses H2 and H4 were rejected. Idealized influence had the strongest effect on employee performance based on path coefficient, followed by inspirational motivation (refer to Figure 2).

### Results of IPMA

**Figure 3:**
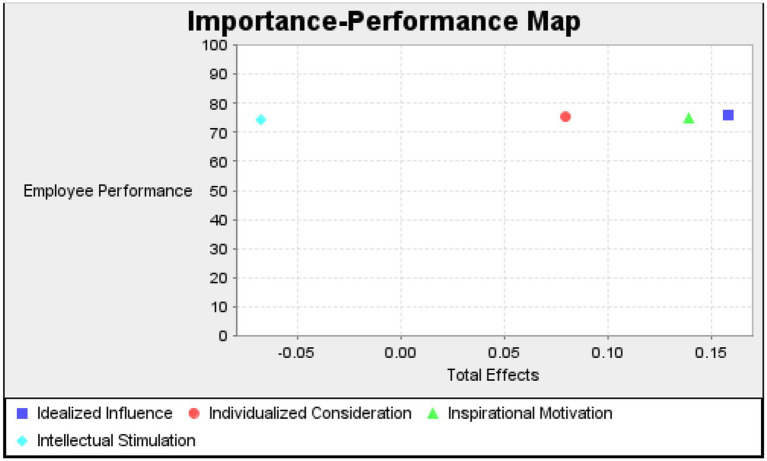
Importance performance map of employee performance.

We continue to use the PLS path model on the same data to determine the importance and performance of these variables. The goal of Importance-Performance Matrix Analysis (IPMA) is to determine the importance of each latent variable in terms of its total impact on each target endogenous construct (performance). Table 5 displays the results, and Figure 3 depicts the “importance-performance map.” Table 5 shows that idealized influence is the most important indicator, followed by individualized consideration, inspirational motivation, and intellectual simulations. While idealized influence is the highest performance indicator, it is followed by inspirational motivation, individualized consideration, and intellectual simulations. Employee performance was found to be significantly influenced by idealized influence and inspirational motivation. However, individualized consideration and intellectual simulations were not significant important and performance indicators of employee performance.

**Table 5:** IPMA results.

Latent variable	Employee performance	
	Total effect (importance)	Index value (performance)
Idealized influence	0.239	75.870
inspirational motivation	0.187	74.779
individualized consideration	0.103	75.282
Intellectual simulations	−0.097	74.212

## Discussion

The fundamental purpose of this research is to investigate the influence of transformational leadership dimensions (idealized influence, individualized consideration, inspirational motivation, and intellectual stimulation) on employee performance in the hospitality industry in Malaysia. The results show that idealized influence and inspirational motivation were significant predictors of employee performance, whereas individualized consideration and intellectual stimulation were not.

Specifically, idealized influence in transformational leadership was the strongest predictor of employee performance. Thus, this study confirms the findings by (Langat et al., 2019; Ma and Yang, 2020; Widodo et al., 2020). Idealized influence, as the current study shows, is the most influential factor in employee performance, and clearly, leaders in the hospitality industry should display determination and a sense of authority while providing a clear vision and sense of mission to their followers. By doing so, they are able to acquire the trust and respect of their subordinates or followers, which in turn, improve their performance. With regard to idealized influence, it is recommended that leaders motivate, stimulate and inspire followers to better performance through a collective sense of mission (Lindawati and Parwoto, 2021). Leaders should also display high moral and ethical standards to be role models for their subordinates (Buil et al., 2019). Leaders going beyond self-interest for the good of the group demonstrate good teamwork within the team, which may improve employee performance, and in turn, achieve organizational goals.

In addition, the findings of H3 reveal that the positive impact of inspirational motivation on employee performance is in line with the studies conducted by (Hasija et al., 2019; Top et al., 2020; Kinya and Eliud, 2021). Leaders in the hospitality industry should inspire their subordinates by expressing a motivating, exciting and clear vision. The needs of the subordinates must be identified by leaders to encourage them to work towards the company’s long-term goals. Additionally, leaders should show a positive outlook on their achievements and goals, and be excited and optimistic in their work to inspire their subordinates to perform better.

As for H2, the hypothesis was rejected since individualized consideration was seen to have no significant influence on employee performance. This is contradictory to the study by Al-Khaled and Fenn (2018), where the researchers stated that the company gained from transformational leadership since it was able to understand its employees’ requirements in order to retain them by managing workload in a friendlier manner, resulting in subordinates feeling satisfied with their work. However, it is supported by Chen et al. (2018), with the researchers stating that leaders who paid attention to their followers on an individual basis caused their followers to be more stressed. Followers, on the other hand, have a moral duty to respond accordingly based on the concept of reciprocation. In reality, perceived personalized consideration is a sign of partiality and unfairness, which can lead to a breakdown in leader-follower relationship.

Hypothesis 4 was rejected as it showed that intellectual stimulation had a significant negative impact on employee performance. Our study is contradictory to the study by Chebon et al. (2019), which states that intellectual stimulation promotes creative thinking. According to Dansereau et al. (1995), by giving intellectual stimulants, managers encourage staff to try new approaches and generate ideas, which can have a substantial influence on performance. As the nature of the job in the hospitality industry is generally repetitive and follows a strict routine, intellectual stimulation may not be relevant to them; however, leaders in other industries may benefit from implementing intellectual stimulation as part of transformational leadership style. For instance, a study conducted by Premi et al. (2020), with special reference to the IT Industry in Chennai, had concluded that intellectual stimulation had a significantly positive impact on the performance of employees. Other researchers may continue to research this dimension on other industries to determine its significance.

### Implications

This research attempts to address multiple gaps in literature and in doing so, makes important implications to academia. Based on the gaps identified in literature, the first implication is that this research links transformational leadership dimensions to employee performance in the Malaysian hospitality industry. As stated by Ahmad et al. (2020), in dealing with the lack of studies on transformational leadership and employee performance, this study has contributed to filling this research gap. Second, as indicated by Ferozi and Chang (2021), though transformational leadership as a whole has a direct or indirect impact on employee performance, it is difficult to locate studies that explore how each dimension of transformational leadership is related to employee performance, and this study investigates how each dimension affects employee performance. Last but not least, although employee performance is a topic that is widely studied around the world and in Malaysia (Basri et al., 2017; Lor and Hassan, 2017), few studies have examined leadership styles in the COVID-19 pandemic context (Kuofie and Muhammad, 2021). As this study was conducted during the pandemic, the results identify how each dimension of transformational leadership affects employee performance during the time. Future researchers may compare the results of this study versus studies made prior to or post-pandemic to discover if there are differences in employees’ perceptions on how the dimensions of transformational leadership affect their performance.

In addition, one of the goals of this project is to give key insights into the Malaysian hospitality business so that the industry may improve employees’ work performance. This study, which focuses on human resource management, might be beneficial to the managers in this industry. Employers can use the information to better understand how to increase employee performance in order to meet the organization’s ultimate goals. In particular, this study will be extremely beneficial to the Malaysian hotel business.

### Limitations and recommendations

There are a few shortcomings in this study. First, because our research was done in Malaysia, it may have been impacted by the Malaysian culture; thus, the applicability of our findings to other nations and cultures remains unknown. Future studies should compare differences across cultures and nations. Second, the data used in this study are cross-sectional. As it is difficult to establish a causal link using a cross-sectional study, future researchers should examine causal relationships using longitudinal investigations. Third, as this study utilizes self-evaluation of overall performance, the results could be lopsided, as people, on the whole, exaggerate their strengths while concealing their shortcomings (Khan et al., 2020). Only employee impressions, rather than genuine leadership and communication actions, were examined, while the viewpoints of communication managers and leaders were not included. To get a more complete, balanced and in-depth understanding as to how leadership and communication factors influence employee change outcomes, future research could use other research strategies, such as interviews, and integrate the viewpoints of change implementation managers and organizational leaders (Yue et al., 2019).

## Conclusion

Within the scope of our study, we carried out an extensive literature review on the many dimensions of transformational leadership and employee performance. We had also discussed on the theory that underpins transformational leadership, as well as the global and Malaysian perspectives on transformational leadership. Our research has led us to the conclusion that the four dimensions of transformational leadership, namely idealized influence, individualized consideration, inspirational motivation, and intellectual stimulation have had partially impact on employee performance in the Malaysian hospitality industry. Based on the findings of this research, it showed that idealized influence and inspirational motivation have a significant impact on the performance of employees. In the hospitality industry, the employee is an essential component in both the profitability and continued existence of hotels. Therefore, leaders set the stage for improved employee performance by providing direction and working to establish a proper foundation for work for both individuals and the organization. Additionally, future research should replicate the current study and focus on aspects of individualized consideration and intellectual stimulation, such as the education and manufacturing industries, which may lead to significantly different findings.

## Data availability statement

The raw data supporting the conclusions of this article will be made available by the authors, without undue reservation.

## Ethics statement

Ethical approval was not provided for this study on human participants because this study was conducted in conformance with Declaration of Helsinki involving humans and all its later amendments. Ethical review and approval were waived for this study due to the fact that it was conducted as a survey analyzing the results of routine questionnaires. The patients/participants provided their written informed consent to participate in this study.

## Author contributions

BT and WW contributed to the conceptualization, study design, data collection, analysis work, and writing the article. BT AS, TS, AV, and SL contributed to editing and preparation for publication. All authors contributed to the article and approved the submitted version.

## Conflict of interest

The authors declare that the research was conducted in the absence of any commercial or financial relationships that could be construed as a potential conflict of interest.

## Publisher’s note

All claims expressed in this article are solely those of the authors and do not necessarily represent those of their affiliated organizations, or those of the publisher, the editors and the reviewers. Any product that may be evaluated in this article, or claim that may be made by its manufacturer, is not guaranteed or endorsed by the publisher.
